# Annotations

**Published:** 1903-02-14

**Authors:** 


### ANNOTATIONS.

### The Tuberculous Soil.

KOCH has made clear the nature of the tuberculous seed. Much conflict of opinion still exists, however, regarding the so-called "tuberculous soil." Some have contended that the interest in the former has led to the importance of the latter being neglected. Notable pronouncements have recently been made regarding the factor of individual susceptibility in phthisis, but still we are far from a clear apprehension of the true value to be given to the predisposing and protecting influences of the several tissues in different individuals. Professor Clifford Allbutt speaking at Glasgow a little time ago, contended that the very word "soil" when applied to the tissues of a tuberculous patient, is a misnomer. He holds that the work of such pioneers as Metchnekoff and Ehrlich and the numerous investigators who have extended and expanded their researches in regard to immunity and what we are accustomed to name "susceptibility," has proved that the bacillus of tubercle, so far from finding in the human body a "soil"—that is a medium more or less proper and conducive to the germination of the seed—"finds, even in the weakest subjects, a medium in which its presence is resented from the beginning, and from which, in a vast majority of cases, it is successfully repelled." Every human being resists the insemination; happily most men discard it at once; some more or less unsuccessful, a few succumb. But a medium which generally prevents insemination, which generally repels germination, which always enters into conflict with the germ, is not well called a "soil."

### The Hypnotic Treatment of Criminals.

THE sale of the so-called relics of Newgate and the demolition of the old prison buildings serve to remind us of the purely repressive and punitive methods adopted in former times, indeed, up to a very recent period, in dealing with those who had been convicted of crime. In striking contrast with the methods of a hundred years ago are those which are being put in operation in scores of prisons at the present time, and in still greater contrast with the views then held are those which are now being preached on every side in regard to criminology. We are now told that the criminal is to a large extent an irresponsible agent of inherited instability, that his mental state is to be regarded as a disease, and that prisons should be regarded as hospitals for the treatment of moral illness rather than as institutions for the punishment of offences against the law. Elmira shows how far practical effect has been given to these notions in one direction, while the remarks made by Sir Oliver Lodge in a recent address to the Society for Psychical Research suggests how far theories may carry men in another. Sir Oliver Lodge said that what was wanted was not force, but intelligent treatment of the mental malady from which the habitual criminal suffered, and he mentioned hypnotic treatment by suggestion as a possible solution of the problem. We need hardly say that the Society which he was addressing takes itself, and all the curious experiences which are related at its meetings, far too seriously to allow us to imagine for a moment that the slightest scintillation of a joke entered into the remarks made by Sir Oliver Lodge, and, indeed, if post-hypnotic suggestion possesses a tithe of the powers often attributed to it, it surely ought to be capable of keeping a man "straight" for 24 hours. It is a curious prospect that rises in one's imagination at the thought. The prison turned into a thieves' doss-house, to which these sufferers from unsoundness of mind in regard to *meum* and *tuum*, would tamely return each evening, and from which they would start out every morning for their day's work after receiving, with a force proportionate to their case, the "suggestion" that they are upright and worthy members of the community, that petty larceny is a thing foreign to their nature, and that they must come in to supper at seven o'clock! And why not? We are asked to accept far more strange things than this as the results of hypnotism, and many of us profess our willingness to believe. If hypnotic suggestion cannot keep a man with a full belly from picking and stealing, what is the good of it?

### Surgeons in the Days of Elizabeth.

AT the present day surgery takes so high a place and so dominates the professional horizon, that it is difficult to realise how subordinate a position the surgeon occupied in early days in all his relations with the physician. As for the apothecary, who was a tradesman selling drugs and compounding prescriptions, it was natural enough that he should occupy a very humble position in comparison with the physician, who at least posed as a man of learning. But that the surgeon, who nowadays so rules the roost, should until recent times have had to bow so low to the physician suggests what is indeed the truth that surgery has in recent times been far more progressive as an art than medicine. In an interesting address on the Elizabethan Revival of Surgery, delivered before a meeting of the Elizabethan Literary Society, Mr. D'Arcy Power tells us that from very early times in England the surgeons had felt it a grievance that they were not allowed to take complete charge of their patients. They were looked upon merely as craftsmen, able indeed to wield the knife and saw, but wholly incapable of ordering medicine or regulating the diet of those upon whom they had operated. To such an extent was this view carried that a physician had to be called on every occasion if more than a trifling change was required in the regimen or medicine, the surgeon being thus kept in quite a subordinate position. This was not unnaturally felt by the better class of surgeons to be an intolerable hardship, and they devoted themselves actively to promote the unity of medicine and surgery. But the power of the physicians was too great, and instead of being able to free themselves they were soon in a worse plight than before, for in 1632 the College of Physicians procured an order in Council with a clause to the effect that "no chirurgeon doe either dismember, trepan the head, open the chest or belly, cut for the stone, or do any great operation with his hand upon the body of any person, but in the presence of a learned physician, one or more of the College or of his Majesty's physicians." Mr. D'Arcy Power says that it was not until after the year 1800 that a hospital surgeon was allowed complete control of his cases.

